# Improved global air quality health index reveals ozone and nitrogen dioxide as main drivers of air-pollution-related acute mortality

**DOI:** 10.1016/j.oneear.2025.101488

**Published:** 2025-11

**Authors:** Wenzhong Huang, Tiantian Li, Pierre Masselot, Rongbin Xu, Antonio Gasparrini, Francesco Sera, Michelle L. Bell, Masahiro Hashizume, Susanne Breitner, Shilu Tong, Haidong Kan, Zhengyu Yang, Yiwen Zhang, Wenhua Yu, Pei Yu, Shuang Zhou, Qinghua Sun, Jingwei Zhang, Eric Lavigne, Joana Madureira, Yue Leon Guo, Vânia Gaio, Shanshan Li, Yuming Guo, MCC Collaborative Research Network

**Affiliations:** 1 Climate, Air Quality Research Unit, School of Public Health and Preventive Medicine, Monash University, Melbourne, VIC, Australia; 2 National Key Laboratory of Intelligent Tracking and Forecasting for Infectious Diseases, National Institute of Environmental Health, Chinese Center for Disease Control and Prevention, Beijing, China; 3 China CDC Key Laboratory of Environment and Population Health, National Institute of Environmental Health, Chinese Center for Disease Control and Prevention, Beijing, China; 4 Environment & Health Modelling (EHM) Lab, Department of Public Health Environments and Society, London School of Hygiene & Tropical Medicine, London, UK; 5 Chongqing Emergency Medical Center, Chongqing University Central Hospital, School of Medicine, Chongqing University, Chongqing, China; 6 School of Public Health and Preventive Medicine, Faculty of Medicine, Nursing and Health Sciences, Monash University, Melbourne, VIC 3004, Australia; 7 Department of Statistics, Computer Science and Applications “G. Parenti,” University of Florence, Florence, Italy; 8 School of the Environment, Yale University, New Haven, CT, USA; 9 Department of Global Health Policy, Graduate School of Medicine, The University of Tokyo, Tokyo, Japan; 10 IBE-Chair of Epidemiology, LMU Munich, Munich, Germany; 11 Institute of Epidemiology, Helmholtz Zentrum München – German Research Center for Environmental Health, Neuherberg, Germany; 12 National Institute of Environmental Health, Chinese Center for Disease Control and Prevention, Beijing, China; 13 School of Public Health and Social Work, Queensland University of Technology, Brisbane, QLD, Australia; 14 Department of Environmental Health, School of Public Health, Fudan University, Shanghai, China; 15 School of Epidemiology & Public Health, Faculty of Medicine, University of Ottawa, Ottawa, ON, Canada; 16 Air Health Science Division, Health Canada, Ottawa, ON, Canada; 17 Department of Environmental Health, Instituto Nacional de Saúde Dr. Ricardo Jorge, Porto, Portugal; 18 EPIUnit – Instituto de Saúde Pública, Universidade do Porto, Porto, Portugal; 19 Laboratório para a Investigação Integrativa e Translacional em Saúde Populacional (ITR), Porto, Portugal; 20 Environmental and Occupational Medicine, National Taiwan University (NTU) College of Medicine and NTU Hospital, Taipei, Taiwan; 21 National Institute of Environmental Health Science, National Health Research Institutes, Zhunan, Taiwan; 22 Graduate Institute of Environmental and Occupational Health Sciences, NTU College of Public Health, Taipei, Taiwan; 23 Department of Epidemiology, National Health Institute Doutor Ricardo Jorge, Lisbon, Portugal; 24 Public Health Research Center, National School of Public Health, NOVA University of Lisbon, Lisbon, Portugal; 25 Comprehensive Health Research Center, NOVA University of Lisbon, Lisbon, Portugal

## Abstract

Ambient air pollutants are leading contributors to global mortality. Despite the well-established risks, most studies have relied on single-pollutant models in limited regions, leaving the combined effects and individual contributions of pollutants unclear, particularly across countries. Here, we integrate daily mortality and air pollutant (nitrogen dioxide [NO_2_], ozone [O_3_], fine particulate matter, and sulfur dioxide) data from 482 cities in 12 countries/territories from 1998 to 2021 to assess the joint mortality risks and identify the main contributing pollutant through an air quality health index of multi-pollutant constrained groupwise additive models (AQHI-Multi). AQHI-Multi outperformed commonly used air quality indices in capturing the overall mortality risks. O_3_ and NO_2_ were the leading contributors (accounting for over 70% across countries/territories), with O_3_’s share increasing slightly to moderately in most countries/territories. These findings highlight the need for developing air quality indices using advanced multi-pollutant models and the emerging global significance of targeted control of O_3_ and NO_2_.

## Graphical abstract

**Graphical abstract:**
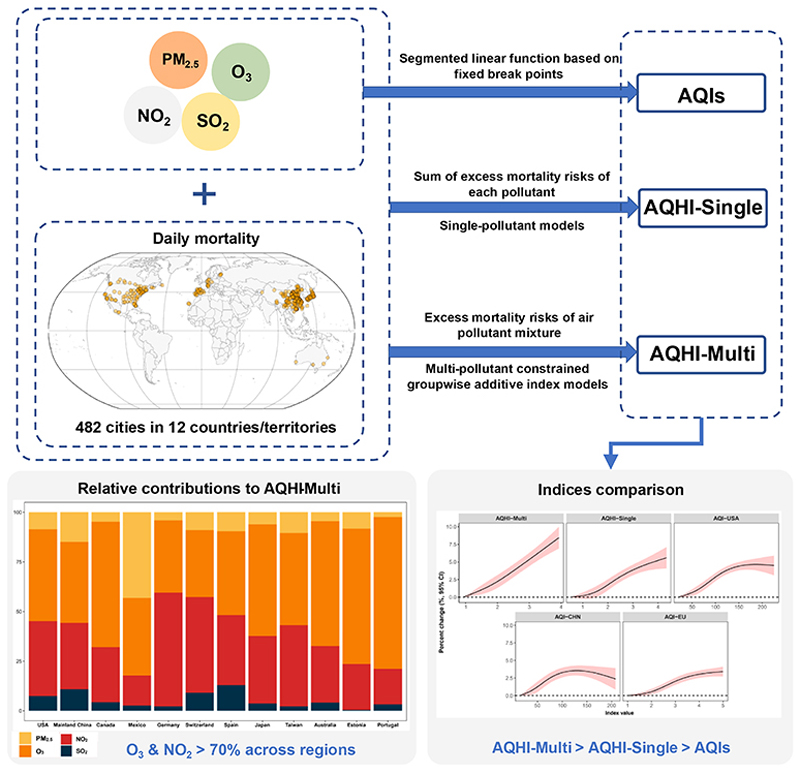

## Introduction

Air pollution is a significant public health concern, with both short- and long-term exposure leading to a wide range of diseases. It has been estimated that air pollution accounted for more than 6.5 million deaths globally each year,^1^ affecting cardiovascular health,^2^ respiratory health,^3^ and other conditions such as diabetes,^4^ obesity,^5^ and neurological disorders.^6^ Despite the substantial and well-established evidence, air pollution remains a leading contributor to the global burden of disease (BoD).^7^ Moreover, the air pollution-related BoD is expected to increase due to climate change and an aging population.^8,9^ It is crucial and urgently needed to develop effective interventions to mitigate the foreseen air-pollution-related BoD and inform the most effective air quality policies.

Accurately conveying real-time health risks of air pollutants and their relative contributions to the public and stakeholders could serve as an effective and important public health strategy in mitigating the air-pollution-related BoD.^10,11^ The development of an intuitive and interpretable index for non-experts that accurately represents the overall health risks of air pollution and the identification of major hazardous air pollutants for stakeholders are crucial for effective risk mitigation.^12^ The Air Quality Index (AQI), originally developed by the US Environmental Protection Agency (EPA) in 1976, is now the most widely applied index to inform the general public of short-term risks of ambient air pollution.^13,14^ However, despite the various guidelines for AQI development in different countries and regions, the AQI is threshold based and calculated as the maximum value of the segmented linear function with fixed breakpoints for each air pollutant (i.e., the maximum individual AQI of each pollutant).^15,16^ Therefore, AQI does not adequately and accurately account for continuous exposure-response functions and the empirical joint health effects of exposure to multiple air pollutants.^16,17^ Importantly, for many pollutants, such as particulate matter and ozone, research indicates the absence of a threshold or safe level below which no adverse health impacts are observed.^18,19^

To address these limitations, the Air Quality Health Index (AQHI), which incorporates the risk estimates of air pollution from empirical epidemiological evidence, was proposed as an improved alternative to the AQI.^16^ However, the currently used AQHI is constructed using the estimates from a single pollutant model that does not account for the presence of other air pollutants.^20^ Considering that people are typically exposed to mixtures of air pollutants rather than a single pollutant in reality, an AQHI calculated using co-effects estimated through a multi-pollutant model strategy may offer greater accuracy in representing the health risks. Additionally, employing a multi-pollutant model allows for the assessment of each pollutant’s contribution to the overall risks, which remains largely unknown.^21^ This will improve our understanding of the health effects of air pollutants across regions characterized by diverse population demographics and varying air-pollution profiles. Such information may also be essential for developing targeted mitigation strategies and informed policy interventions that prioritize reducing the major hazardous air pollutants, thereby effectively and affordably decreasing the air-pollution-related BoD.

Here, we integrated city-level mortality and monitored air-pollution data over the last two decades from 12 countries/territories across four continents, spanning diverse socioeconomic contexts, including the largest developed country (the United States [US]) and the largest developing country (China). We aimed to develop an improved AQHI using an advanced multi-pollutant constrained groupwise additive model (AQHI-Multi) to more accurately capture the overall mortality risks from short-term exposures to ambient air pollutants and to quantify the relative contributions of individual pollutants in different countries/territories. We observed that the AQHI-Multi exhibited a non-threshold, generally strongest and most linear relationship with mortality compared to commonly used air quality indices. Ozone (O_3_) and nitrogen dioxide (NO_2_) together contributed over 70% of the AQHI-Multi, with a slight to moderate temporal increase in the relative contribution of O_3_ in most countries/territories. The findings support the potentially superior accuracy of multi-pollutant model-based indices in representing and conveying the mortality risks of air-pollution mixtures on a multi-country scale and highlight the increasing global priority of implementing targeted controls for O_3_ and NO_2_ to reduce the air-pollution-related BoD.

## Results

### Descriptive statistics

**Figure 1:**
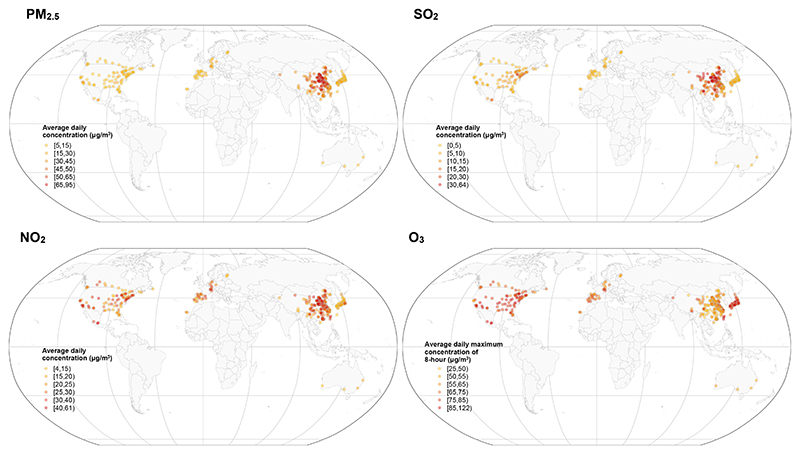
Spatial distribution of the 482 cities in 12 countries/territories; the average daily mean concentration of PM_2.5_, SO_2_, and NO_2_; and the daily maximum concentration of 8-h O_3_ during the data-collection period NO_2_, nitrogen dioxide; O_3_, ozone; PM_2.5_, particulate matter with aerodynamic diameter ≤ 2.5 μm; SO_2_, sulfur dioxide.

We analyzed 19.8 million deaths from 482 cities within 12 countries/territories (Figure 1 and Table S1). The populations in these cities experienced a broad range of air-pollution levels, with city-specific mean concentration (μg/m^3^) ranging from 5.3 in Canada to 94.0 in Mainland China for daily average particulate matter with aerodynamic diameter ≤2.5 μm (PM_2.5_), from 29.2 in Mainland China to 121.2 in Taiwan for daily maximum 8-h average O_3_, from 4.8 in Japan to 60.8 in Mainland China for daily average NO_2_, and from 0.9 in Estonia to 61.2 in Mainland China for daily average sulfur dioxide (SO_2_) (Table S1). Large countries/territories (Mainland China and the US) included more heterogeneous air-pollution levels across cities. Detailed descriptive statistics of the deaths and air pollution for each country/territory are summarized in Table S1. The wide range of air-pollution levels and geographic distribution are illustrative of regions characterized by different climates and socioeconomic development levels, from developed regions in North America and northern Europe to developing areas in Eastern Asia (Figure 1).

### Mortality risks associated with ambient air pollutants

**Figure 2:**
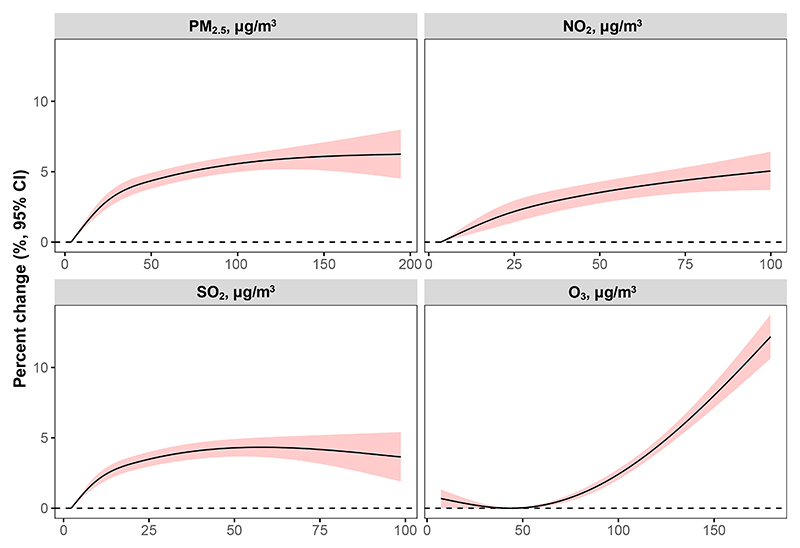
The overall exposure-response curve of mortality risks, i.e., percentage change in mortality, with each of the air pollutants PM_2.5_, SO_2_, NO_2_, and O_3_ The shaded area indicates the 95% confidence interval (CI). NO_2_, nitrogen dioxide; O_3_, ozone; PM_2.5_, particulate matter with aerodynamic diameter ≤ 2.5 μm; SO_2_, sulfur dioxide.

Figure S1 shows the overall lag-response pattern of the mortality risks associated with PM_2.5_, SO_2_, NO_2_, and O_3_ in the week after exposure (lag 0–7 days). The mortality risks increased consistently after exposure to higher levels of PM_2.5_, SO_2_, NO_2_, or O_3_, generally peaking in the first 2 days after exposure (lag 0–1 day) and persisting up to 3 days (lag 0–2 days). As the elevated mortality risks tended to be statistically insignificant and minimal 3 or more days after exposure, in subsequent analysis we applied a 3-day moving average for PM_2.5_, SO_2_, NO_2_, or O_3_ concentration, which represented the average of the same and the previous 2 days (lag 0–2 days), to capture the short-term adverse effects of air pollution. Figure 2 shows overall cumulative exposure-response curves of mortality risks with each air pollutant. As the PM_2.5_, NO_2_, and SO_2_ exposure levels increased, the mortality risks demonstrated a supra-linear and monotonic increase. These curves remained similar after excluding cities without data on all-cause mortality (Figure S2) or after imputing missing air-pollution data (Figure S3). Nearly identical results were also observed when data after the year 2019 were excluded (Figure S4).

### Associations of various air quality indices with mortality

**Figure 3:**
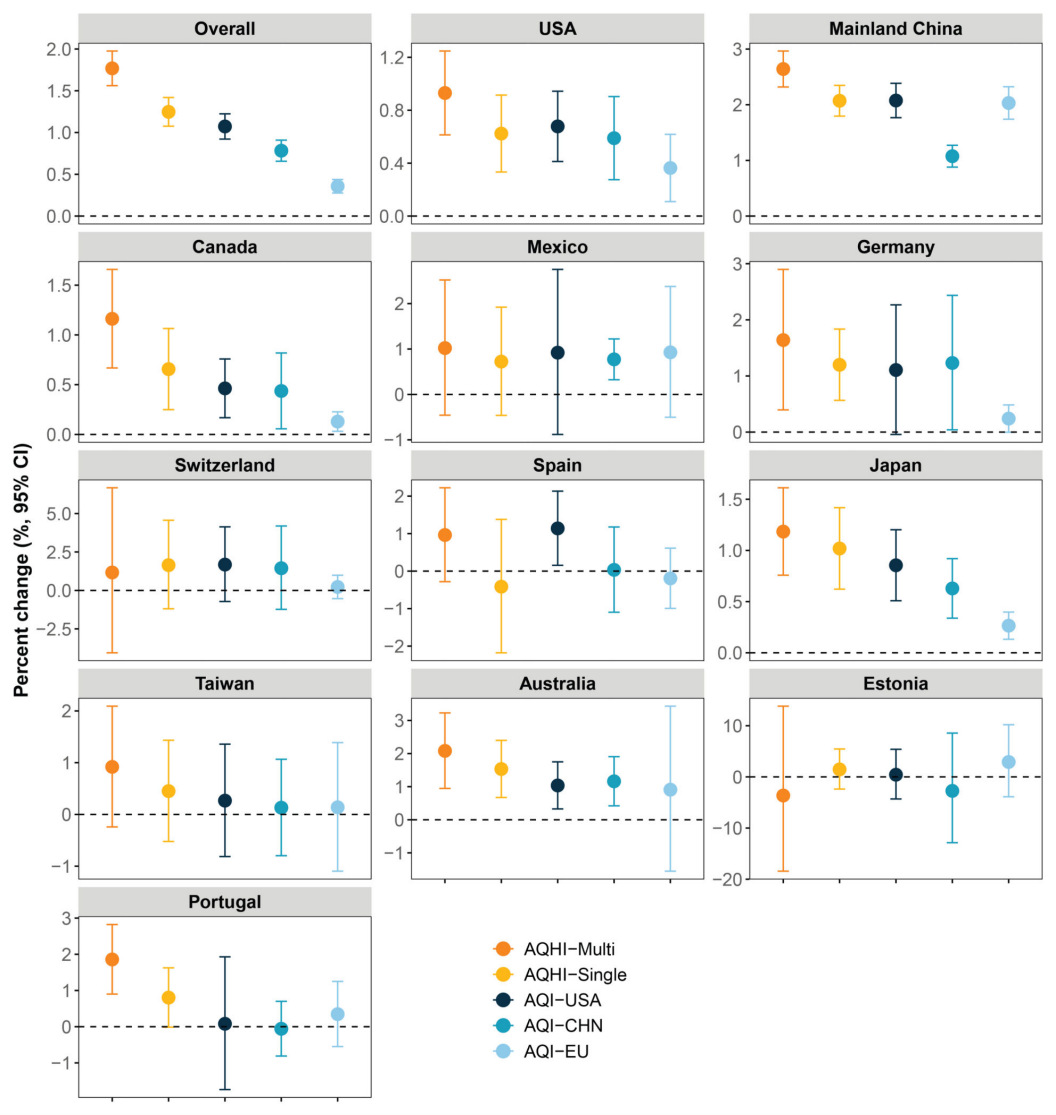
The overall and country/territory utility of AQI-USA, AQI-EU, AQI-CHN, AQHI-Single, and AQHI-Multi, presented as the percentage change in mortality with 95% CI, for each interquartile increase in the index The utility was examined in testing data using the parameters estimated based on training data. The early 70% of the data were selected as the training data and the remaining 30% as the testing data for each city. AQI-USA, the US Environmental Protection Agency’s air quality index; AQI-EU, the European Environment Agency’s air quality index; AQI-CHN, the Chinese Ministry of Ecology and Environment’s air quality index; AQHI-Single, the air quality health index based on the single-pollutant model; AQHI-Multi, the air quality health index based on the multi-pollutant CGAIM; CGAIM, constrained groupwise additive index model; CI, confidence interval.

The overall and country/territory-specific utility of various types of indices of air quality is depicted in Figure 3. Overall, AQHI based on a multi-pollutant model (i.e., AQHI-Multi) represented the highest mortality risk, demonstrating the strongest association with unobserved mortality (i.e., mortality outcomes in the separate test dataset), followed by AQHI based on a single-pollutant model (i.e., AQHI-Single), the US version of AQI (AQI-USA), the China version of AQI (AQI-CHN), and the European Union version of AQI (AQI-EU). At the country level, the relatively higher utility of AQHI-Multi was also observed in most study countries/territories, especially for the US, Mainland China, Canada, Portugal, and Japan, compared to other indices of air quality. The utility of the different indices of air quality was robust to models with various specifications (Figure S5) or using different proportions of training data (Figure S6). Similarly, the index-response curves showed that AQHI-Multi had the strongest, most of the study countries/territories. Notably, the relative contribution weight of O_3_ showed a slight to moderate increase over time in most of the study countries/territories, with the most pronounced increases observed in Mainland China, Canada, Spain, and Estonia.

## Discussion

**Figure 4:**
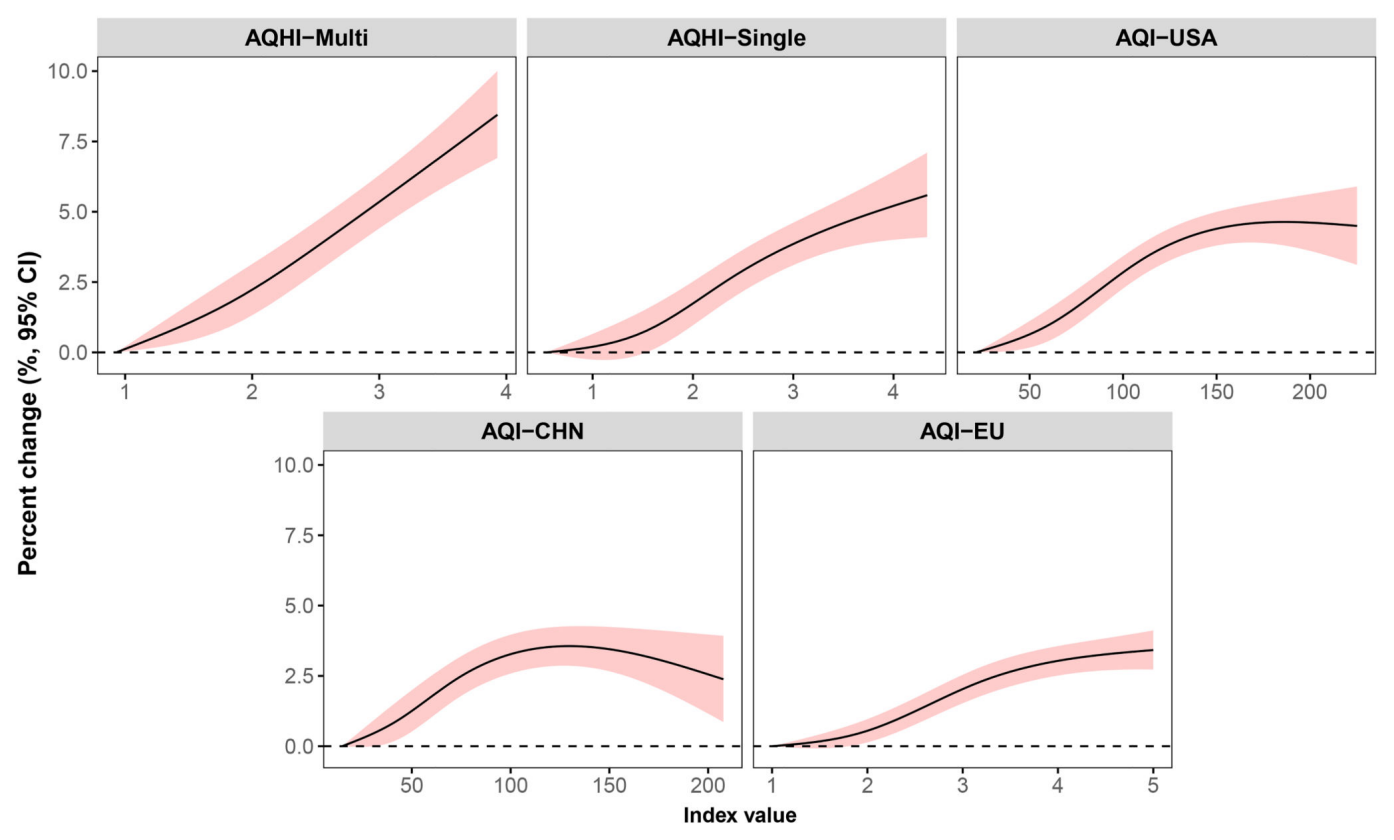
The index-response curve of various air quality indices with mortality risks The shaded area indicates the 95% CI. The curves were fitted using the indices in testing data constructed based on the parameters derived from the training data. The early 70% of the data were selected as the training data and the remaining 30% as the testing data for each city. AQI-USA, the US Environmental Protection Agency’s air quality index; AQI-EU, the European Environment Agency’s air quality index; AQI-CHN, the Chinese Ministry of Ecology and Environment’s air quality index; AQHI-Single, the air quality health index based on the single-pollutant model; AQHI-Multi, the air quality health index based on the multipollutant CGAIM; CGAIM, constrained groupwise additive index model; CI, confidence interval.

Despite the well-established elevated most linear, and non-threshold relationship with mortality risk compared to the other indices (Figure 4). As the index value increased, the air-pollution-related mortality risks rose proportionally for AQHI-Multi. In contrast, the mortality risks tended to level off or even decrease with the increase of AQHI-Single and AQIs.

### Spatiotemporal patterns of AQHI-Multi and pollutant contributions

**Figure 5:**
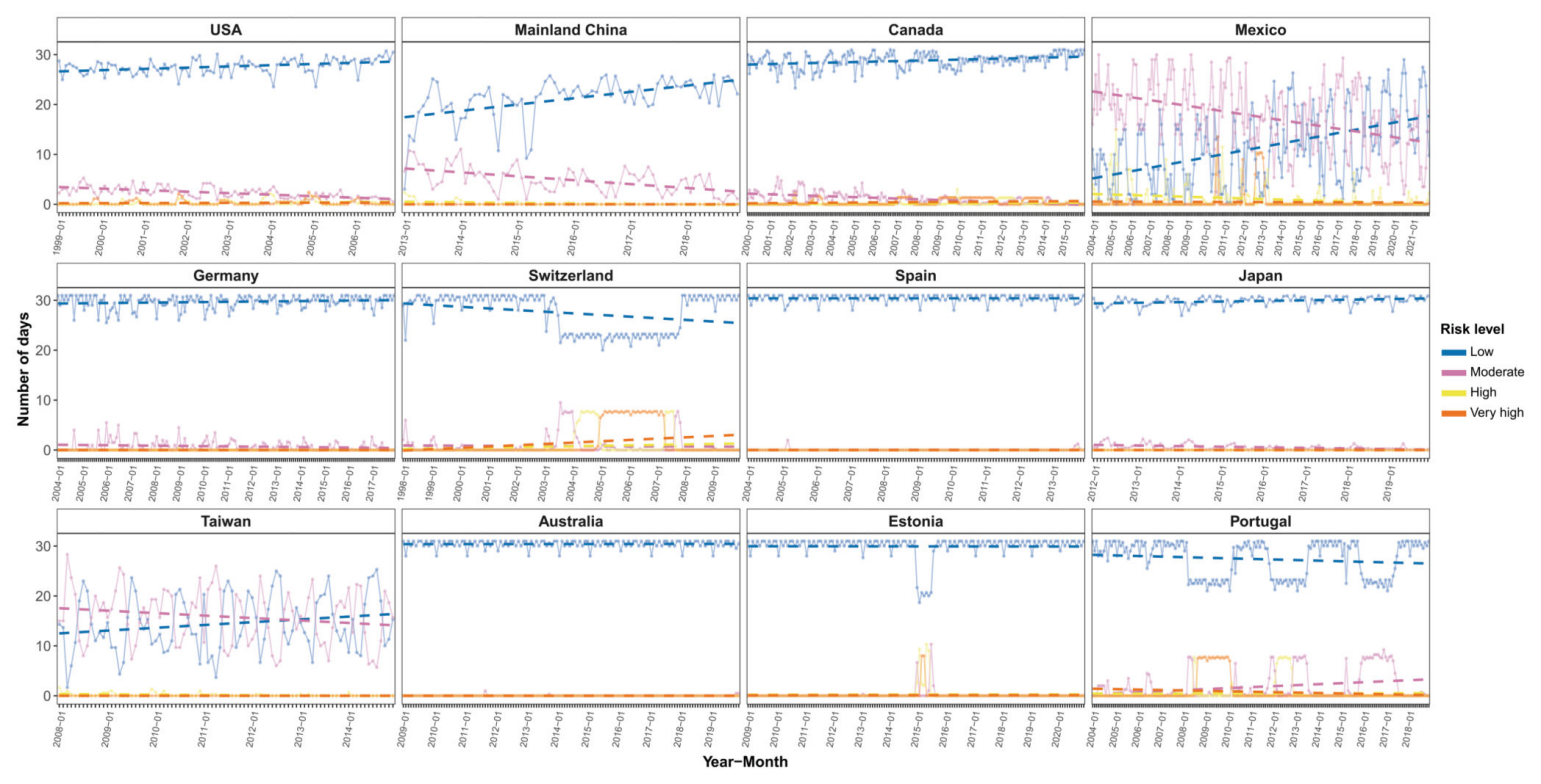
Temporal evolution of the monthly number of days of each air quality risk category based on AQHI-Multi in each study country/territory during the study period The health risks from air pollution in a day were classified into four categories based on the AQHI-Multi values: low (1–3), moderate (4–6), high (7–10), and very high (>10). The dashed lines represent the linear trend. AQHI-Multi, the air quality health index based on the multi-pollutant CGAIM; CGAIM, constrained groupwise additive index model.

**Figure 6:**
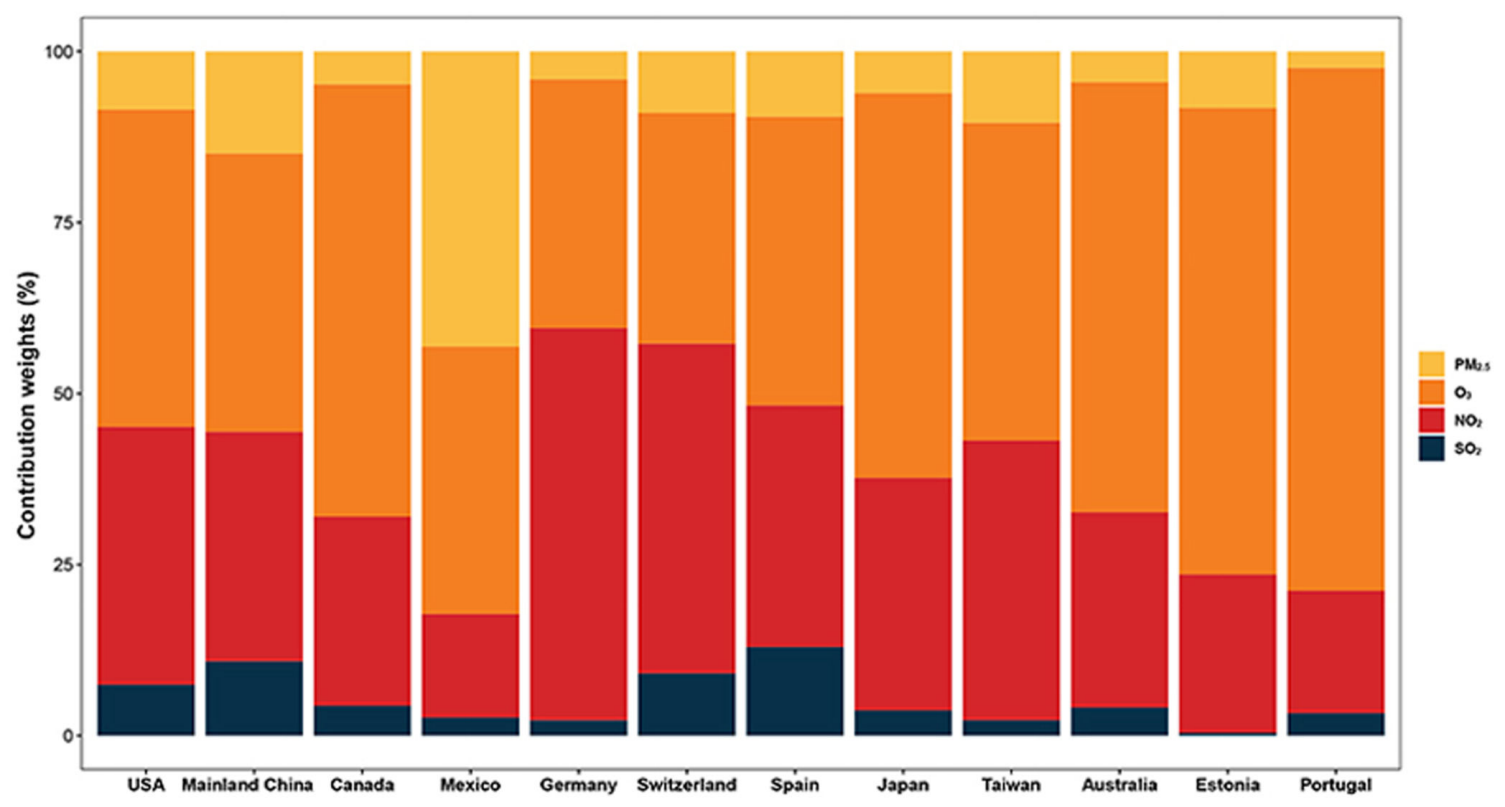
Overall relative contribution weight in percent of PM_2.5_, SO_2_, NO_2_, and O_3_ to AQHI-Multi in each country/territory over the study period AQHI-Multi, the air quality health index based on the multi-pollutant CGAIM; CGAIM, constrained groupwise additive index model; NO_2_, nitrogen dioxide; O_3_, ozone; PM_2.5_, particulate matter with aerodynamic diameter ≤ 2.5 μm; SO_2_, sulfur dioxide.

The final constructed index of AQHI-Multi for each country/territory during the study period is shown in Figure 5, presented as the monthly number of days in each air quality risk category based on the AQHI-Multi value. The risk parameters for calculating the AQHI-Multi in each study country/territory are shown in Table S2. The number of low-risk days (i.e., AQHI-Multi ≤3) in a month generally decreased in Switzerland and Portugal during the study period while increasing in Mainland China, Mexico, and Taiwan (Figure 5). The trends remained relatively constant over time in the US, Canada, Germany, Spain, Japan, Australia, and Estonia. The relative contributions of each air pollutant (PM_2.5_, O_3_, SO_2_, and NO_2_) to the AQHI-Multi in each country/territory are depicted in Figure 6. The specific contributions to an elevated AQHI-Multi varied across air pollutants and countries/territories. Generally, O_3_ and NO_2_ were the leading air pollutants that contributed to a higher value of AQHI-Multi in different countries/territories, followed by PM_2.5_ and SO_2_. The contributions of O_3_ were particularly high in Canada, Japan, Australia, Estonia, and Portugal, accounting for more than 50% of the air-pollution-related joint mortality risks represented by AQHI-Multi. In contrast, NO_2_ was the main air pollutant driving the increase in AQHI-Multi in Germany and Switzerland. The contributions of SO_2_ were consistently and comparatively limited across countries.

When viewed on a temporal scale, the contributions of various air pollutants to AQHI-Multi showed seasonal variations (Figure S7). Generally, relatively higher contributions of O_3_ and NO_2_ were observed in summer and winter, respectively, in mortality risks associated with short-term exposures to air pollutants, their joint mortality risks and respective contributions remain unclear, especially across diverse geographic regions. This reduces the accuracy of quantifying and communicating overall real-world mortality risks, constrains mechanistic understanding of pollutant-specific health impacts, and impedes the formulation of evidence-based, targeted mitigation strategies. Based on multi-country data and an advanced multi-pollutant model, we developed an improved air quality index (i.e., AQHI-Multi) by incorporating empirical population risks from co-exposures to air pollutants to more accurately present and convey the overall mortality risks associated with ambient air pollution. AQHI-Multi exhibits a higher utility in representing the overall mortality risks associated with air-pollution exposure compared to the currently used AQIs and AQHI based on the single-pollutant model or considerations of multiple pollutants without accounting for their joint effects. The risk contributions of PM_2.5_, SO_2_, NO_2_, and O_3_ varied across countries/territories. Generally, O_3_ and NO_2_ were the leading air pollutants that contributed most to AQHI-Multi across countries/territories. A slight to moderate increase in the relative contribution of O_3_ was observed over time in most of the study countries and territories.

Air-pollution warning indicators serve as an important tool for communicating health risks to the public and mitigating the health burden from air-pollution exposure.^14^ However, the currently most widely used air-pollution indicator, the AQI, is calculated solely based on the air-pollution concentrations. This does not explicitly account for the population vulnerability. Specifically, populations with different characteristics exhibit varying mortality risks even when exposed to the same pollution levels. To date, studies on the development of air-pollution risk indicators remain scarce, especially on a multi-country scale.^14,22^ By considering both air-pollution levels and population vulnerability, the AQHI was developed as an improved alternative to the AQI through the inclusion of health risk estimates of air pollutants derived from epidemiological evidence. Further, people are exposed to multiple pollutants rather than a single air pollutant in real life. In this study, we improved the AQHI by considering the joint effects of air pollutants using an advanced multi-pollutant model. These improvements were evidenced by our validity analysis, where AQHI-Multi exhibited higher utility in presenting the air-pollution-related mortality risks, followed by AQHI-Single and AQIs. A non-threshold and approximately linear index-response relationship was exclusively observed for AQHI-Multi in the test dataset, in contrast to other indices. This suggested that AQHI-Multi was a more sensitive indicator that accurately reflected the health risks equivalent to its value at different levels of air pollution. By comparison, AQHI-Single and AQIs may lack effectiveness in accurately conveying the risks of air-pollution-related mortality, particularly during periods of low or high air-pollution levels. Our findings are consistent with previous studies on AQHI establishment that were largely based on a single-pollutant model or restricted to a single area or region in Mainland China.^20,23–25^ Similarly, these studies also suggested a stronger association of AQHI with health outcomes compared to AQI. This study advanced previous AQHI research by moving beyond single-pollutant models and geographically limited analyses, employing a multi-pollutant model across diverse countries and territories that could quantify both joint mortality risks and individual pollutant contributions more accurately by accounting for previously overlooked potential non-linear and combined effects. These advances provide a more comprehensive and accurate basis for health risk communication and the development of effective response strategies for ambient air pollution.

Based on the improved approach for AQHI constructions, we calculated AQHI-Multi and characterized the temporal trends in air-pollution-related joint mortality risks, as well as the contributions of different air pollutants across various countries and territories. The diverse patterns observed in these trends indicated great variations in the temporal changes of air-pollution-related mortality risks across different countries/territories. Currently, evidence on the temporal trends in the overall mortality risk from air-pollutant mixtures from these countries/territories remains limited. This constrains the ability to detect changes or improvements in public health outcomes over time, undermines the assessment of long-term effectiveness of air quality policies, and hampers timely identification of emerging risks from pollutant mixtures. We found that the monthly number of low-risk days (i.e., AQHI-Multi ≤3) exhibited the most significant increase in Mainland China. This highlights the great significance of China’s devoted efforts during the past decade since the introduction of the “Atmosphere Ten Articles” policy in 2013 to dramatically reduce the air-pollution level, which could prevent hundreds of thousands of annual attributable premature deaths.^26^

We further examined the relative contributions of each air pollutant to the mortality risks associated with short-term exposure to air-pollutant mixtures, as represented by the AQHI-Multi, in various countries. Despite the different magnitude of contribution to the mortality risks for various air pollutants in different countries/territories, O_3_ and NO_2_ made a prominent contribution to the AQHI-Multi in most of the study countries/territories. By comparison, the contribution of SO_2_ was consistently limited across different study countries/territories. While there is substantial evidence to support the adverse associations of air pollution with human health, studies with a quantitative assessment of the specific contribution of each air pollutant compared to other pollutants are scarce, especially on a multi-country scale.^27,28^ Previous studies on health risk assessment of air pollutants were largely based on a single- or two-pollutant model that did not consider the co-existence of multiple pollutants, partially to avoid multi-collinearity due to the high correlations among air pollutants.^20,21^ The indices developed by such approaches likely bias population health risk estimates, thereby reducing the accuracy and effectiveness of public health risk assessment and communication and potentially leading to suboptimal or misdirected air quality interventions. By comparison, multi-pollutant models such as constrained groupwise additive index models (CGAIMs) can account for the potential multi-collinearity and provide the joint risk estimate of co-exposures to multiple pollutants as well as the relative weight of each pollutant.^29^ Leveraging an advanced multi-pollutant CGAIM, the developed AQHI-Multi advances beyond prior single- or limited-pollutant risk assessments by more accurately quantifying the relative importance of each pollutant from a perspective of public health. This enhanced understanding supports the development of targeted air quality management strategies to cost-effectively reduce the substantial disease burden currently attributable to air pollution. Based on the real-time informed index of AQHI-Multi and the relative contribution weight from each air pollutant, limited resources can be prioritized based on the pollutants that currently contribute most significantly to health risks.

Our findings were generally consistent with our previous investigation in a metacity (Guangzhou) in Mainland China, which also used a multi-pollutant model and detected that O_3_ and NO_2_ were the major air pollutants contributing to elevated morbidity (outpatient visits and hospital admissions) and mortality risks.^20^ The current study supports this finding on a multi-country scale and highlights the leading role of O_3_ and NO_2_ in contributing to air-pollution-related acute mortality in different countries/territories with diverse geographic and sociodemographic contexts. Furthermore, compared with the previous multi-pollutant model-based AQHI establishment approaches (e.g., Bayesian multi-pollutant models),^20,23,30–32^ the AQHI-Multi developed using CGAIM in this study enables intuitive interpretation and flexible integration of operational requirements and prior knowledge in a computationally efficient manner. This makes it more adaptable for local health authorities and environmental agencies to develop region-, population-, and outcome-specific air quality health indices while avoiding the computationally intensive procedures (e.g., Markov chain Monte Carlo sampling). The observed dominant role of O_3_ and NO_2_ could be partly attributed to the highly reactive and potent oxidant properties as well as their typically higher concentrations in urban environments, especially in developed regions.^33,34^ These factors can provoke more immediate inflammatory responses compared to PM_2.5_ and SO_2,_ including the triggering of asthma exacerbations and acute cardiovascular events.^35–38^ Furthermore, we observed that the relative contribution of O_3_ to AQHI-Multi and air-pollution-related premature mortality has increased over time in most of the study countries and territories. This trend could be mainly driven by the increasing level of O_3_ exposure, especially under a warming climate. Climate change has resulted in longer and hotter summers, which extend the ozone season and increase peak O_3_ concentrations.^39^ Targeted strategies need to be developed to reduce the pollution levels of O_3_ and NO_2_ to more effectively and affordably mitigate the heavy disease burden associated with air pollution.

This study had multiple strengths. By using a large population-based dataset across countries/territories, we were able to establish an improved AQHI that robustly captures the mortality risks associated with complex air-pollutant mixtures across regions with diverse geographic and sociodemographic contexts and to examine its utility and applicability in different settings. For outcome data, we used 19.8 million mortality observations with relatively high spatiotemporal resolution (city- and daily-level) from a well-established framework of the Multi-City Multi-Country (MCC) network that has been widely used in previous studies.^40–45^ This provided high statistical power and broad representativeness within a validated epidemiological framework. For exposure data, we used reliable monitoring of air-pollution concentrations from local authorities within each country/territory, thereby reducing local measurement error that could bias risk estimates, in contrast to the modeled exposure data used in most previous studies. For the modeling strategy, we used a well-established time-series model^46,47^ and an advanced multi-pollutant model,^29^ which have been illustrated above.

However, some limitations must also be acknowledged. For the US, we only had data from 1999 to 2006, which limited the generalizability of our findings to the current conditions in the US. Additionally, our data collection and analysis were performed at the city level, assuming uniform air-pollution exposures for individuals within each city. This non-differential exposure misclassification tends to systematically bias the risk estimates toward the null, which indicates our results are more likely to be conservative, and the findings on the greater utility of AQHI-Multi compared to other air quality indices are unlikely to be substantially affected.^48,49^ Furthermore, the AQHI-Multi in the present study was formulated based on mortality data from urban areas, limiting the applicability to inform the morbidity risks (e.g., outpatient visits) and risks for populations in suburban or rural areas. However, considering that AQHIs are commonly applied in urban contexts and mortality data are generally more readily available, this approach enhances the applicability and reproducibility of our findings. Moreover, although we quantified the varying contribution of different air pollutants to health risks across regions, we did not have data on pollutant chemical composition or emission sources, which precluded us from delineating the specific mechanisms underlying these spatial variations (e.g., potential differences in toxicity). Finally, the limited number of study locations in some countries (e.g., Mexico and Germany) restricted the statistical power to differentiate the utility of different health indices of air quality in these countries. However, results from larger countries/territories with many more study locations (e.g., the US, Mainland China, and Canada) consistently supported a higher accuracy of AQHI-Multi in representing the air-pollution-related mortality risks compared to other indices. Therefore, this limitation may be more likely to have contributed to a more conservative interpretation of our findings. Future studies could apply and extend the AQHI-Multi formulation strategy to other countries and health outcomes to validate and complement our findings.

In summary, we found that the mortality risks related to air pollution were more accurately represented by the AQHI constructed using multi-pollutant models (i.e., CGAIMs) than by currently used air quality indices on a multi-country scale. O_3_ and NO_2_ were consistently identified as the main pollutants contributing to the short-term mortality risks from ambient air pollution, with the relative contribution of O_3_ showing slight to moderate increases over the past two decades in most of the studied countries/territories. The results of this study emphasize the urgent need for local health authorities and environmental agencies to integrate advanced multi-pollutant models to more effectively capture and communicate air-pollution-related health risks. In addition, more mitigation efforts and enhanced targeted strategies should be devoted to responding to the emerging health impacts of O_3_ and NO_2_.

## Methods

### Data sources

We obtained health and environmental data from the database of the MCC Collaborative Research Network, which has been described in detail in our previous work.^40,42^ In brief, daily data on mortality, monitoring of ambient air pollution, temperature, and relative humidity were obtained from local authorities within each country/territory. The current analysis was limited to cities available for four major air pollutants: daily average concentration of PM_2.5_, SO_2_, NO_2_, and daily maximum 8-h average O_3_ (482 cities in 12 countries/territories, with an overall study period ranging from 1998 to 2021) (Table S1). PM_10_ and carbon monoxide (CO) were not included because (1) PM_10_ exhibits a high correlation with PM_2.5_, and PM_2.5_ already encompasses the primary adverse effects associated with particulate matter^50^; and (2) the adverse public health impact of ambient CO is comparatively modest as the concentrations of CO consistently remain well below the air quality standards established in various countries and territories.^20,51^ Daily PM_2.5_, SO_2_, NO_2_, and daily maximum 8-h average O_3_ had an overall missing rate of 4.0%, 5.3%, 1.9%, and 2.1%, respectively. Days with missing pollutant measurements were excluded during the analysis. In 23 out of the 482 cities, all-cause mortality data were not available and, instead, mortality from non-external causes (International Classification of Diseases, 9th Revision [ICD-9], codes 0–799 or ICD-10, codes A0−R99) was collected to represent all-cause mortality.^40–42^

### Statistical analysis

#### Single-pollutant model

In the single-pollutant model, we adopted a standard two-stage analytical framework to estimate the associated mortality risk for each air pollutant (PM_2.5_, SO_2_, NO_2_, and O_3_).^40,42,52^ In the first stage, a quasi-Poisson regression distributed lag non-linear model, accounting for the possible overdispersed daily mortality counts, was applied to model the lag-response and exposure-response relationships of air-pollution concentration with mortality in each city and for each air pollutant. To account for the non-linear and delayed effects of air pollution, the air-pollutant concentration was modeled with a distributed non-linear and lag term up to 7 days after exposure, with a natural cubic spline function of two internal knots placed at the 25th and 75th concentration percentiles and two internal knots (plus an intercept) equally placed on the log scale of lag days, respectively.^40,53^ Natural variations in the mortality (i.e., long-term trends, seasonal and weekly patterns) were adjusted by using a natural cubic spline of time with 7 degrees of freedom (df) per year and including a dummy variable of day-of-week indicator in the model.^40,54,55^ Potential confounding effects by weather conditions were also controlled by including 8-day moving averages of ambient temperature and relative humidity with natural cubic splines of 6 and 3 df, respectively, in the model.^40,56,57^ We tested these modeling choices in the sensitivity analysis.

In the second stage, the city-specific effect estimates from the first stage were pooled using random-effects meta-analysis to characterize the overall lag-response and exposure-response relationships between mortality and air pollutants. The pooled risk estimates are presented as the percentage change (with 95% confidence intervals) in daily mortality per 10 μg/m^3^ increase in air-pollutant concentrations.

#### Multi-pollutant model

In the multi-pollutant model, we applied a two-stage analytical framework similar to the above single-pollutant analysis. In the first stage, we applied CGAIM to estimate the joint mortality risks of air-pollutant mixture (PM_2.5_, SO_2_, NO_2_, and O_3_) and to find the optimal weights for each air pollutant that represent potentially adverse effects in each city.^58^ The development and methodology of CGAIM has been detailed in our previous work.^58^ In brief, compared to the traditional single-pollutant model, CGAIM includes all exposures of interest simultaneously in the model as a group within which index weights and the potentially non-linear association between the index and health are estimated. Given the complexity of integrating multiple pollutants, CGAIM additionally allows constraints on both the weights and the association to yield meaningful and stable indices that reflect the overall health impact of all exposures of interest. The constraints in CGAIM allow the integration of additional information reflecting prior assumptions about the risk estimates as well as the operational requirements to ensure both identifiability and a better interpretability of the resulting indices. More specifically, we implemented a constraint that all relative contribution weights of air pollutants in the mixture were ≥0 and summed to 1, and that the relationship of the air-pollutant mixture with mortality was monotone increasing.^58^ The CGAIM was adjusted for the same confounders as those in the single-pollutant model (i.e., natural variations in the mortality and weather conditions).

In the second stage, the effect estimates generated from the CGAIM in each city were pooled using random-effects meta-analysis to obtain overall and country/territory-specific joint risk estimates of the air-pollutant mixture as well as the relative contribution weights of each air pollutant.

### Air quality indices formulation

For each country/territory, we calculated the AQHI-Single and AQHI-Multi using the corresponding country/territory-specific effect estimates from the above single- and multi-pollutant models, respectively. Based on previous studies,^16,20^ we first derived death risk functions (DRFs) related to air pollution using the following formulas:

(Equation 1) $$\text{DRF-Single}\;=100\sum_{k\;=1\;}^{p}\left(e^{\beta_{kc}X_{kti}}-1\right),$$

(Equation 2) $$\text{DRF-Multi}\;=\beta_{c}\sum_{k\;=\;1}^{p}W_{kc}X_{kti},$$

where DRF-Single and DRF-Multi represent the DRF calculated based on the risk parameters from the single-pollutant and multi-pollutant models, respectively. In Equation 1 for DRF-Single, *β*_*kc*_ denotes the risk estimate of air pollutant *k* in country/territory *c* where city *i* is located, and *X*_*kti*_ denotes the concentration (μg/-m^3^) of air pollutant *k* on day *t* in city *i*. Therefore, DRF-Single can also be interpreted as the total excess mortality risks associated with air pollutant 1 to *p*. In Equation 2 for DRF-Multi, the air pollutants are considered as a whole, and *β*_*c*_ denotes the risk estimate of the air-pollutant mixture in country/territory *c*; *W*_*kc*_ refers to the joint mortality risk and relative weight of air pollutant *k* in country/territory *c* where city *i* is located.

To create an understandable and comparable metric and facilitate the risk communications, we further scaled the DRF to index values from 1 to 10+ in a way that the index value of 3 corresponds to the DRF derived for the concentrations where the World Health Organization short-term air quality guideline values in 2021 were met.^59,60^ DRF-Single and DRF-Multi were scaled to AQHI-Single and AQHI-Multi, respectively. A greater AQHI value indicates higher mortality risks associated with short-term exposure to air pollutants. The health risks from air pollution could be classified into four categories based on the AQHI values: low (1–3), moderate (4–6), high (7–10), and very high (>10).^61^

For comparisons, we also calculated a series of currently commonly used AQIs according to the guidelines in different countries and regions. Specifically, we calculated the AQI-USA (ranging from 0 to 500+), AQI-EU (0–6), and AQI-CHN (0–300+) based on the guidelines from the US Environmental Protection Agency,^62^ the European Environment Agency,^63^ and the Chinese Ministry of Ecology and Environment,^64^ respectively. Though having different numerical scales compared to AQHIs (0–10+), a higher AQI value also indicates a greater health risk from air pollution.

### Air quality indices utility

To check the utility and effectiveness in representing mortality risks of different air quality indices, we conducted a validation analysis based on a train-test split.^20,23^ Specifically, for each city, we used the early 70% of data as the training set and the subsequent 30% as the testing set. The data were partitioned into two continuous parts based on time, with one preceding the other, because, in practice, the AQHIs were developed using continuous data from a specific historical period and applied to current and future scenarios. The two-stage single- and multi-pollutant analyses were conducted using the training dataset to generate parameters, which were used to calculate AQHI-Single and AQHI-Multi with the testing dataset. AQI-USA, AQI-EU, and AQI-CHN were also calculated with the testing dataset. For each index, we then estimated and compared the percent change in mortality for each interquartile range (IQR) increase in the index, as well as the index-response relationships of various indices with mortality, using the standard two-stage time-series analysis described above.^20,23^ Specifically, in the first stage, for each city in the test set, we modeled the mortality risks associated with each IQR increase in the index using a quasi-Poisson regression model, with adjustments consistent with those in the main model (i.e., natural variations in mortality, temperature, and relative humidity). We used overall and country/territory-specific IQRs for each index to account for substantial variability in the numerical ranges of the indices and ensure the comparability of the effect estimates across cities and indices. To model potential non-linearity, we conducted additional city-specific models in which the linear index term was replaced with a non-linear term represented by a natural cubic spline function, with two knots positioned at the 25th and 75th percentiles of the index distribution, averaged across all cities in the test set.^20,25,65^ Then, in the second stage, these city-specific effect estimates were pooled using random-effects meta-analysis to derive overall and country/territory-level results. An index with a higher estimate was considered to be more strongly associated with mortality and to have greater utility in representing and predicting mortality risks.

### Sensitivity analysis

To test the robustness of our results, we conducted a series of sensitivity analyses by (1) excluding the cities without all-cause mortality data; (2) imputing missing air-pollutant values using a natural spline function based on other available daily values for each city; (3) excluding the data after the year of 2019 to assess the extent of COVID-19’s potential impact; (4) employing different model specifications, which include altering the df for time (5, 6, 8, or 9 per year) to adjust for temporal trends, df for temperature (3, 4, 5, or 7), the length of the averaging window for temperature and relative humidity adjustments (3 or 21 days), additional adjustment for public holiday, and not adjusting for the relative humidity; and (5) using a different proportion (50%, 60%, 70%, or 80%) of data as the training set.

All analyses were performed using the R language and environment (version 4.1.3),^66^ with the R packages “dlnm”^67^ and “cgaim”^58^ for the single- and multi-pollutant model, respectively. The random-effects meta-analysis was performed using the R package “mixmeta.”^47^

## Resource Availability

### Lead contact

Requests for further information and resources should be directed to and will be fulfilled by the lead contact, Yuming Guo (yuming.guo@monash.edu).

## Materials availability

This study did not provide any novel materials.

## Supplementary Material

Supplemental information can be found online at https://doi.org/10.1016/j.oneear.2025.101488.

Appendix

## Data Availability

All data used in our study were obtained from the MCC Collaborative Research Network (https://mccstudy.lshtm.ac.uk/) under a data-sharing agreement and cannot be made publicly available. Researchers can refer to MCC participants, who are listed in the author list of our study, for information on accessing the data for each country. Demonstrative codes are available at https://github.com/PierreMasselot/cgaim. Any additional information required to reanalyze the data reported in this paper is available from the lead contact upon request.

## Science For Society

Ambient air pollution is a leading threat to premature mortality in populations world-wide. However, current risk assessments and communications often overlook the combined exposure to multiple air pollutants. Most existing measures rely on single- or two-pollutant assessments, while a consistent quantification of the joint effects and individual pollutant contributions across countries and regions remains scarce.

Our study shows that air quality indices developed using multi-pollutant epidemiological models could more accurately capture the short-term mortality risks than currently used indices do. Ozone and nitrogen dioxide are the main pollutants driving these risks, with ozone’s impact growing in most of the studied countries/territories over the past two decades. This improved index can help toward better monitoring, understanding, and communication of air-pollution health risks, enabling more targeted strategies to mitigate the associated substantial disease burden.

## Highlights

- An advanced multi-pollutant model-based air quality health index was developed
- The index outperforms current indices in capturing acute mortality of air pollution
- O_3_ and NO_2_ are the main pollutants driving the risk index across regions
- O_3_ shows rising contributions to the index in most studied countries/territories
