# Supplementary material for: Improved global air quality health index reveals ozone and nitrogen dioxide as main drivers of air-pollution-related acute mortality
Source: One Earth. Author manuscript; Available in PMC 2025 Dec 8. (PMC7618329; doi:10.1016/j.oneear.2025.101488)
Supplement: Appendix [file EMS209693-supplement-Appendix.pdf]

One Earth, Volume *8*

Supplemental information

Improved global air quality health index reveals ozone and nitrogen dioxide as main drivers of air-pollution-related acute mortality

Wenzhong Huang, Tiantian Li, Pierre Masselot, Rongbin Xu, Antonio Gasparrini, Francesco Sera, Michelle L. Bell, Masahiro Hashizume, Susanne Breitner, Shilu Tong, Haidong Kan, Zhengyu Yang, Yiwen Zhang, Wenhua Yu, Pei Yu, Shuang Zhou, Qinghua Sun, Jingwei Zhang, Eric Lavigne, Joana Madureira, Yue Leon Guo, Vânia Gaio, Shanshan Li, Yuming Guo, and MCC Collaborative Research Network

Supplementary Material

Table of Contents

**Table S1**	Summary of the study periods, number of cities and deaths, and air pollution in 12 study countries/territories.
**Table S2**	The risk parameters for calculating AQHI-Multi in each study country/territory.
**Figure S1**	Overall lag effects of air pollution on mortality, presented as the percentage change (with 95% CI) in mortality for each 10 $\mu g/m^3$ increase in $PM_{2.5}$, SO_2, NO_2 and O_3, respectively, along lag 0–7 days.
**Figure S2**	The overall exposure-response curve of mortality risks (percentage change in mortality) with each of the air pollutants ($PM_{2.5}$, SO_2, NO_2 and O_3) by excluding those cities without data on all-cause mortality or not (n = 23).
**Figure S3**	The overall exposure-response curve of mortality risks (percentage change in mortality) with each of the air pollutants ($PM_{2.5}$, SO_2, NO_2 and O_3) by imputing missing air pollution data with a natural spline function. The shaded area indicates the 95% CI.
**Figure S4**	The overall exposure-response curve of mortality risks (percentage change in mortality) with each of the air pollutants ($PM_{2.5}$, SO_2, NO_2 and O_3) by excluding the data after the year of 2019. The shaded area indicates the 95% CI.
**Figure S5**	The overall and country/territory utility of AQI-USA, AQI-EU, AQI-CHN, AQHI-Single and AQHI-Multi, presented as the percentage change (with 95% CI) in mortality for each interquartile increase in the index. The utility was examined in test data using the parameters estimated based on training data. The previous 70% of data points (days) were selected as the training data and the remaining 30% as test data for each city. The utility of each index was examined in models with different specifications.
**Figure S6**	The overall and country/territory utility of AQI-USA, AQI-EU, AQI-CHN, AQHI-Single and AQHI-Multi, presented as the percentage change (with 95% CI) in mortality for each interquartile increase in the index. The utility was examined in test data using the parameters estimated based on training data. The previous 50%, 60%, 70% and 80% data points (days) were selected as the training data and the remaining as test data, respectively.
**Figure S7**	Temporal evolution of monthly relative contribution weight (%) of $PM_{2.5}$, SO_2, NO_2 and O_3 to AQHI-Multi in each country/territory during the study period.

Table S1. Summary of the study periods, number of cities and deaths, and air pollution in 12 study countries/territories.

Country/territory	Period	No. of locations	No. of deaths	Average daily concentration, μg/m^3 (range)			
				$PM_{2.5}$	O_3	NO_2	SO_2
Australia	2009-2019	4	406,780	7.4 (6.3, 8.5)	54.4 (52.6, 56.9)	14.0 (10.5, 16.3)	4.5 (3.2, 6.2)
Canada	2000-2015	24	1,852,630	8.0 (5.3, 12.6)	74.1 (56.7, 86.1)	20.9 (8.7, 34.4)	5.6 (1.1, 17.6)
Estonia	2009-2020	3	29,732	6.8 (5.4, 8.1)	52.8 (50.6, 55.6)	8.7 (5.4, 11.4)	2.5 (0.9, 5.3)
Germany	2004-2017	6	598,126	15.0 (14.0, 16.3)	60.4 (55.8, 66.8)	28.5 (20.6, 38.8)	3.4 (2.3, 4.7)
Japan	2012-2019	47	3,000,018	12.8 (8.3, 17.4)	81.7 (65.9, 92.2)	16.9 (4.8, 37.1)	4.7 (1.0, 15.8)
Mainland China	2013-2018	302	5,305,615	53.9 (15.1, 94.0)	59.2 (29.2, 81.9)	36.2 (9.7, 60.8)	22.2 (5.2, 61.2)
Mexico	2004-2021	4	2,319,773	23.0 (10.7, 31.7)	90.1 (58.8, 114.0)	40.6 (22.3, 51.1)	13.8 (8.0, 18.3)
Portugal	2004-2018	4	457,322	10.3 (6.7, 12.5)	75.5 (62.3, 85.1)	13.2 (4.9, 30.6)	2.8 (1.0, 6.0)
Spain	2004-2013	19	438,720	11.4 (6.2, 21.6)	67.7 (53.9, 83.6)	26.8 (6.7, 45.9)	5.2 (2.5, 9.6)
Switzerland	1998-2009	4	96,267	19.6 (16.9, 24.1)	75.1 (72.5, 76.8)	36.7 (25.6, 49.2)	5.7 (4.5, 7.3)
Taiwan	2008-2014	3	443,680	33.9 (26.9, 41.5)	109.7 (102.0, 121.2)	36.8 (32.7, 41.0)	11.6 (8.6, 16.8)
USA	1999-2006	62	4,897,628	12.8 (6.6, 17.8)	81.0 (54.1, 100.9)	28.3 (7.1, 55.7)	9.5 (2.2, 25.3)
Overall	–	482	19,846,291	38.4 (5.3, 94.0)	66.1 (29.2, 121.2)	31.6 (4.8, 60.8)	16.4 (0.9, 61.2)

Abbreviations: NO_2, nitrogen dioxide; O_3, ozone; $PM_{2.5}$, particulate matter with aerodynamic diameter ≤ 2.5 μm; SO_2, sulfur dioxide.

Table S2. The risk parameters for calculating AQHI-Multi in each study country/territory.

Country/territory	β	Weight			
		O_3	NO_2	$PM_{2.5}$	SO_2
USA	0.461492	0.163383	0.406878	0.186303	0.244987
Mainland China	0.369991	0.271273	0.390781	0.120648	0.217224
Canada	0.260250	0.240068	0.376358	0.176678	0.197011
Mexico	1.602055	0.141817	0.125953	0.666762	0.064572
Germany	0.454158	0.161232	0.576651	0.077006	0.185050
Switzerland	0.112429	0.123404	0.393743	0.014522	0.486412
Spain	0.283568	0.113884	0.261331	0.164185	0.478551
Japan	0.334496	0.161705	0.515834	0.118336	0.202327
Taiwan	0.614174	0.205363	0.545607	0.157722	0.094601
Australia	0.561653	0.241052	0.437401	0.129761	0.191261
Estonia	0.068773	0.249117	0.491619	0.239529	0.029868
Portugal	0.410705	0.421279	0.442066	0.011501	0.131862

Note: the risk parameters were the country/territory-level pooled parameters from the multi-pollutant CGAIM. Abbreviations: AQHI-Multi, the air quality health index based on the multi-pollutant CGAIM; CGAIM, constrained groupwise additive index model; NO_2, nitrogen dioxide; O_3, ozone; $PM_{2.5}$, particulate matter with aerodynamic diameter ≤ 2.5 μm; SO_2, sulfur dioxide.

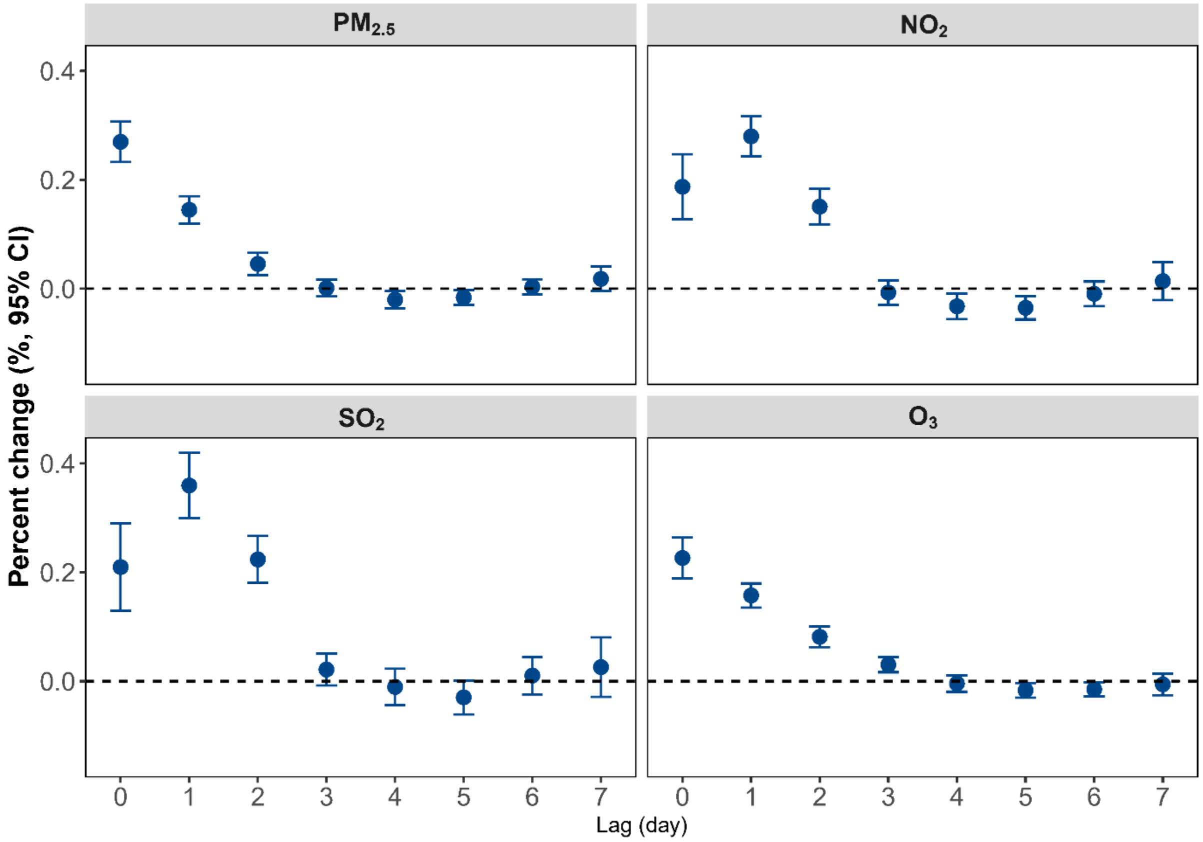

Figure S1. Overall lag effects of air pollution on mortality, presented as the percentage change (with 95% CI) in mortality for each 10 μg/m^3 increase in $PM_{2.5}$, SO_2, NO_2 and O_3, respectively, along lag 0–7 days. Abbreviations: CI, confidence interval; NO_2, nitrogen dioxide; O_3, ozone; $PM_{2.5}$, particulate matter with aerodynamic diameter ≤ 2.5 μm; SO_2, sulfur dioxide.

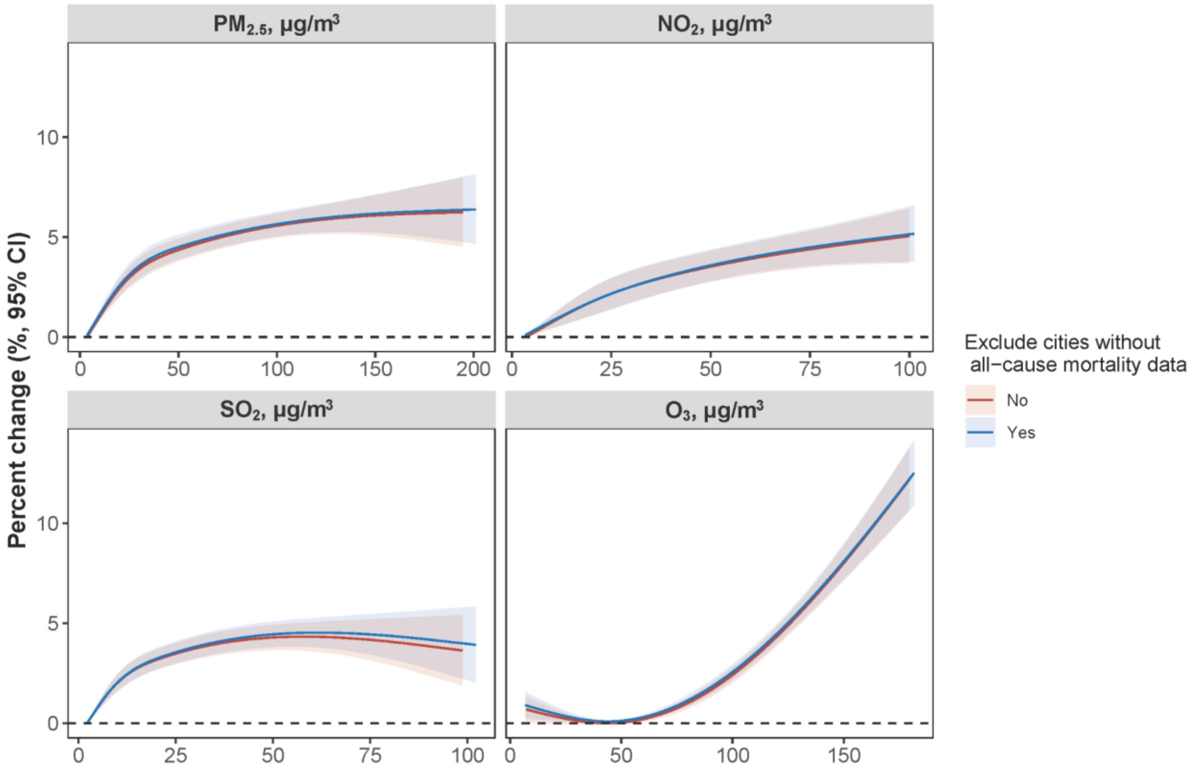

Figure S2. The overall exposure-response curve of mortality risks (percentage change in mortality) with each of the air pollutants ($PM_{2.5}$, SO_2, NO_2 and O_3) by excluding those cities without data on all-cause mortality or not (n = 23). The shaded area indicates the 95% CI. Abbreviations: CI, confidence interval; NO_2, nitrogen dioxide; O_3, ozone; $PM_{2.5}$, particulate matter with aerodynamic diameter ≤ 2.5 µm; SO_2, sulfur dioxide.

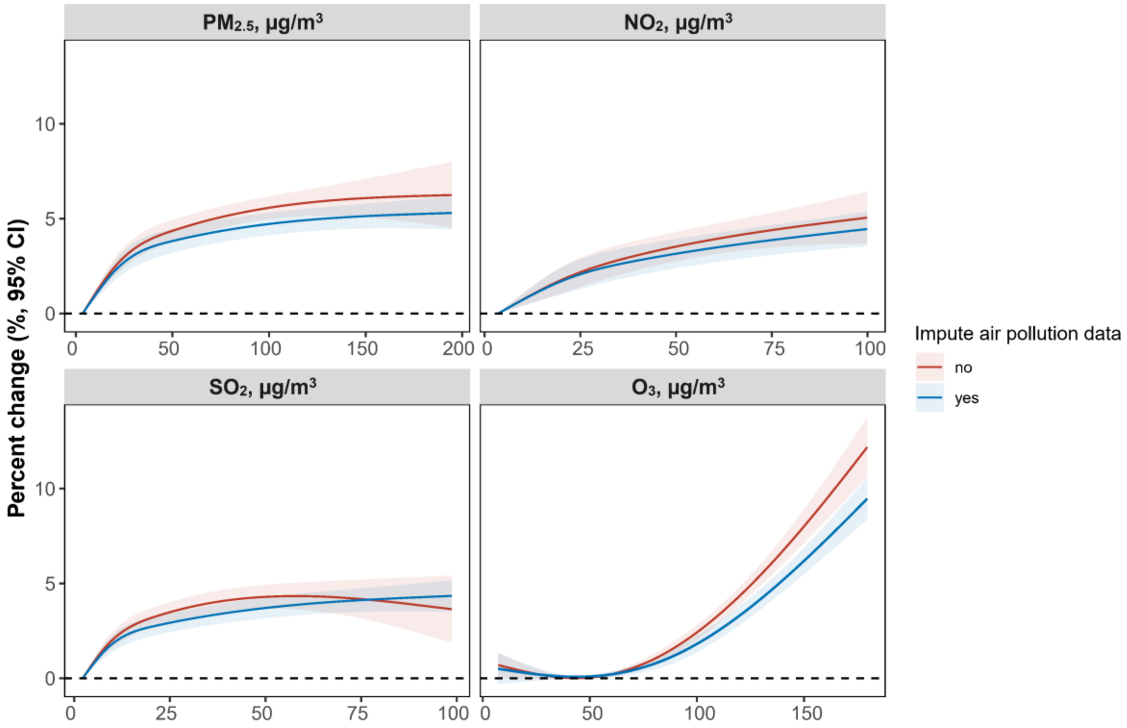

Figure S3. The overall exposure-response curve of mortality risks (percentage change in mortality) with each of the air pollutants ($PM_{2.5}$, SO_2, NO_2 and O_3) by imputing missing air pollution data with a natural spline function. The shaded area indicates the 95% CI. Abbreviations: CI, confidence interval; NO_2, nitrogen dioxide; O_3, ozone; $PM_{2.5}$, particulate matter with aerodynamic diameter ≤ 2.5 µm; SO_2, sulfur dioxide.

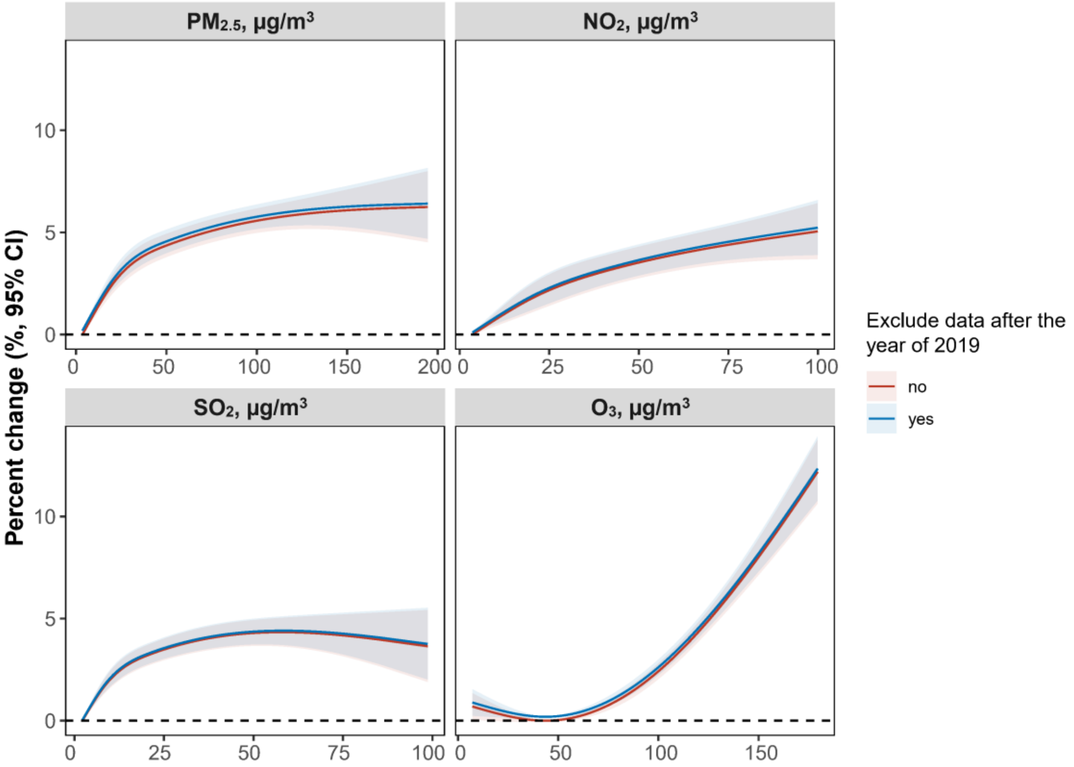

Figure S4. The overall exposure-response curve of mortality risks (percentage change in mortality) with each of the air pollutants ($PM_{2.5}$, SO_2, NO_2 and O_3) by excluding the data after the year of 2019. The shaded area indicates the 95% CI. Abbreviations: CI, confidence interval; NO_2, nitrogen dioxide; O_3, ozone; $PM_{2.5}$, particulate matter with aerodynamic diameter ≤ 2.5 μm; SO_2, sulfur dioxide.

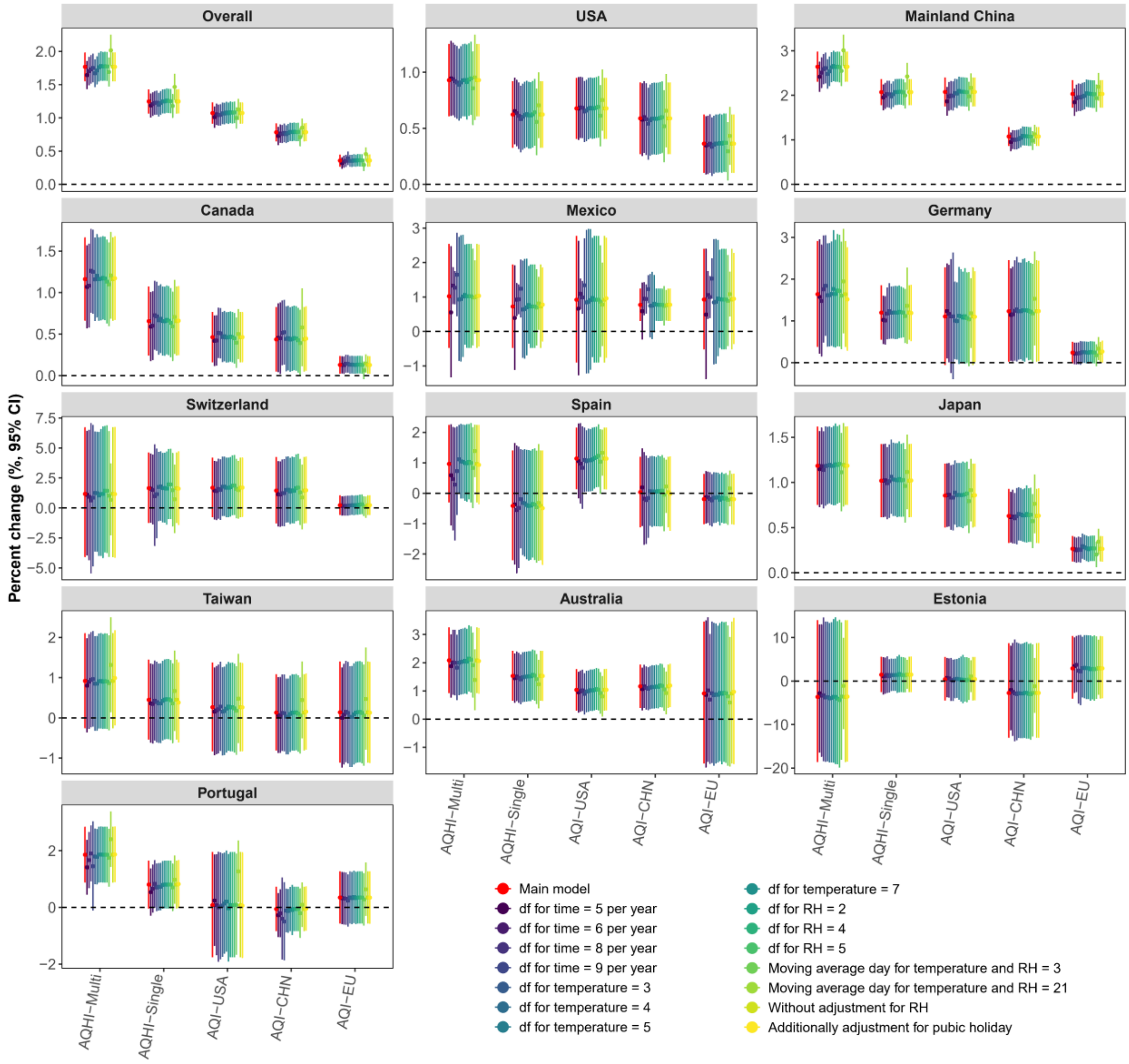

Figure S5. The overall and country/territory utility of AQI-USA, AQI-EU, AQI-CHN, AQHI-Single and AQHI-Multi, presented as the percentage change (with 95% CI) in mortality for each interquartile increase in the index. The utility was examined in test data using the parameters estimated based on training data. The previous 70% of data points (days) were selected as the training data and the remaining 30% as test data for each city. The utility of each index was examined in models with different specifications. Abbreviations: AQI-USA, the U.S. Environmental Protection Agency's air quality index; AQI-EU, the European Environment Agency's European air quality index; AQI-CHN, the Chinese Ministry of Ecology and Environment's air quality index; AQHI-Single, the air quality health index based on the single-pollutant model; AQHI-Multi, the air quality health index based on the multi-pollutant CGAIM; CGAIM, constrained groupwise additive index model; CI, confidence interval; df, degree of freedom; RH, relative humidity.

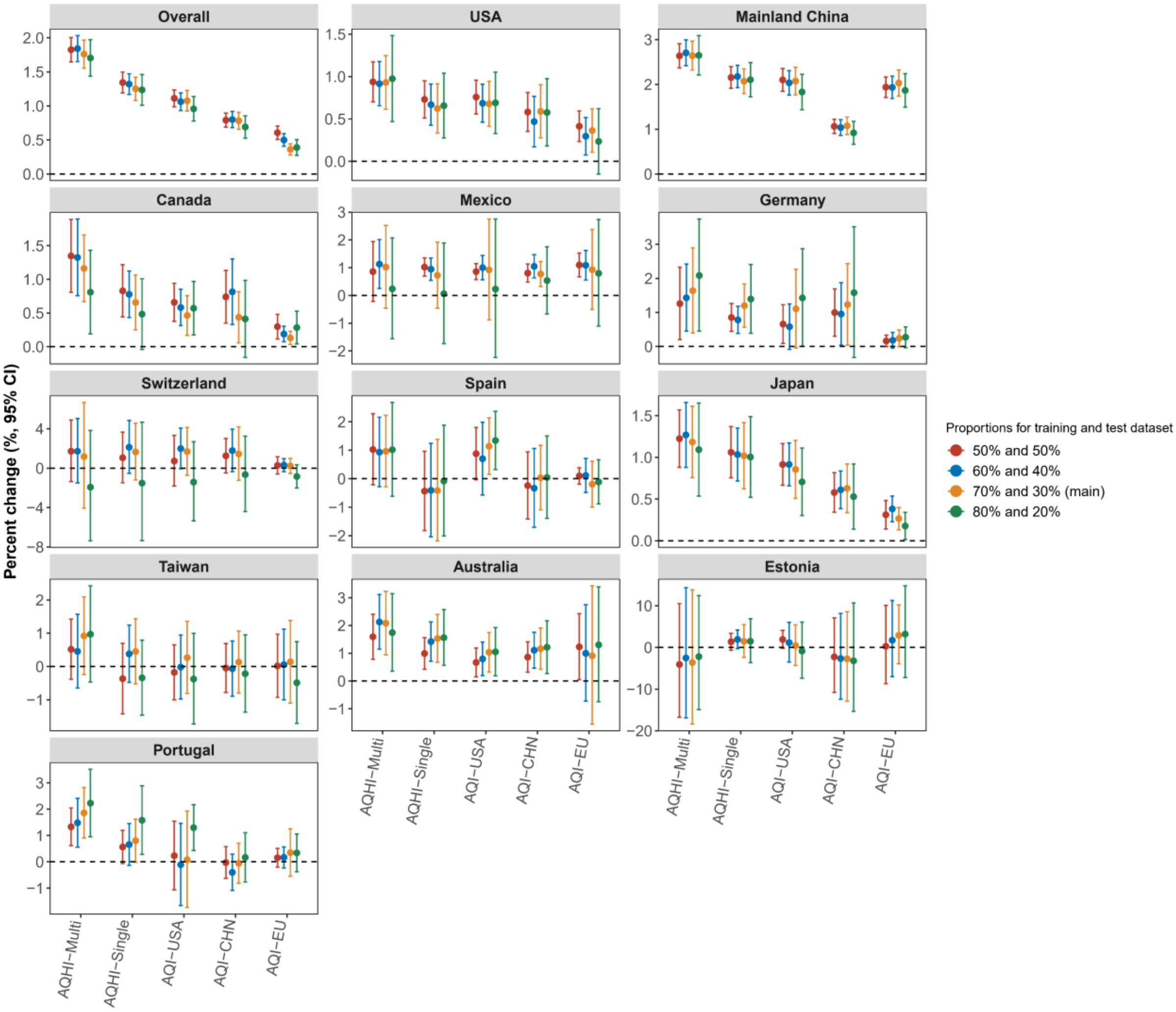

Figure S6. The overall and country/territory utility of AQI-USA, AQI-EU, AQI-CHN, AQHI-Single and AQHI-Multi, presented as the percentage change (with 95% CI) in mortality for each interquartile increase in the index. The utility was examined in test data using the parameters estimated based on training data. The previous 50%, 60%, 70% and 80% data points (days) were selected as the training data and the remaining as test data, respectively. Abbreviations: AQI-USA, the U.S. Environmental Protection Agency's air quality index; AQI-EU, the European Environment Agency's European air quality index; AQI-CHN, the Chinese Ministry of Ecology and Environment's air quality index; AQHI-Single, the air quality health index based on the single-pollutant model; AQHI-Multi, the air quality health index based on the multi-pollutant CGAIM; CGAIM, constrained groupwise additive index model; CI, confidence interval; df, degree of freedom; RH, relative humidity.

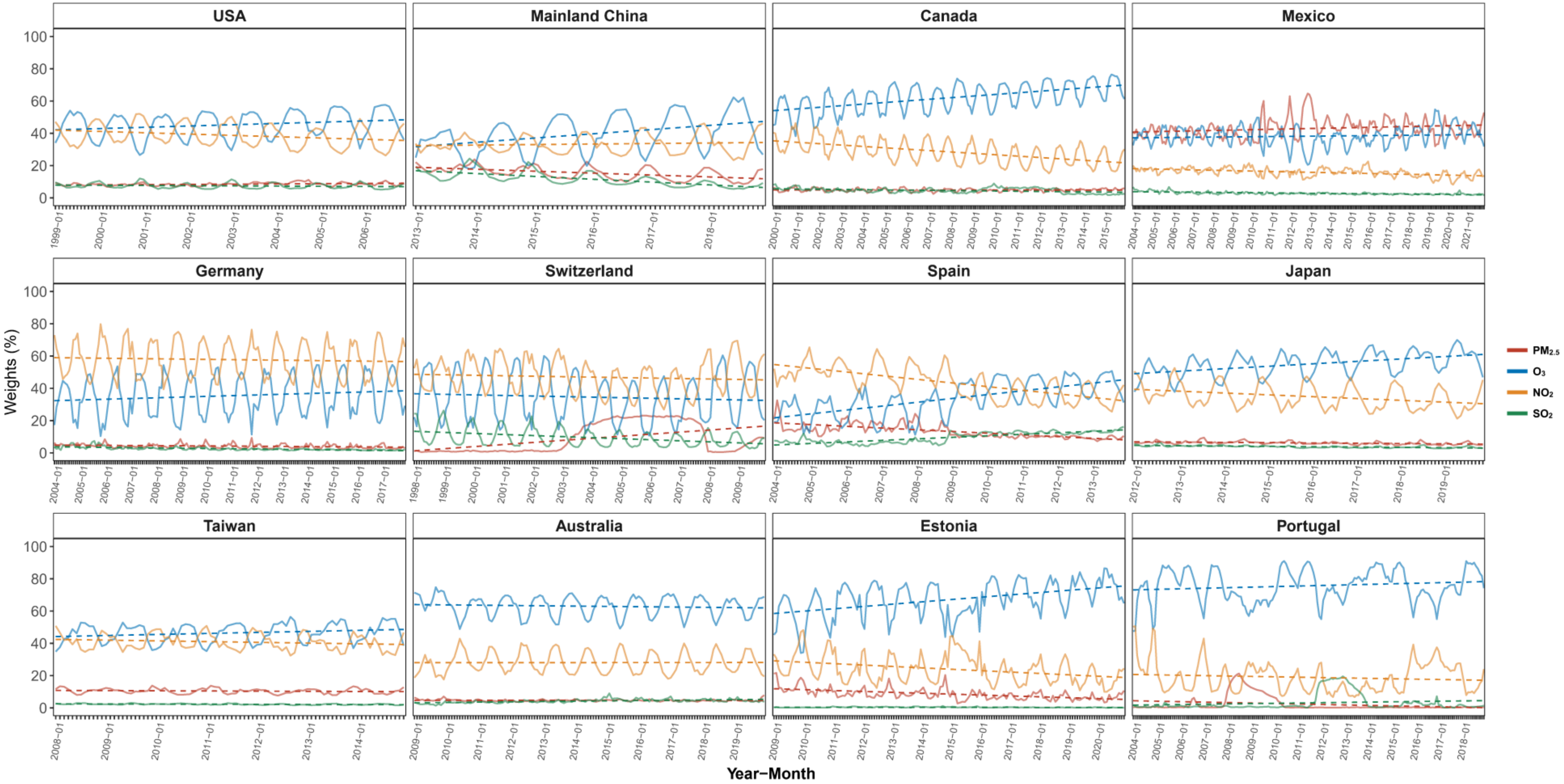

Figure S7. Temporal evolution of monthly relative contribution weight (%) of $PM_{2.5}$, SO_2, NO_2 and O_3 to AQHI-Multi in each country/territory during the study period. Abbreviations: AQHI-Multi, the air quality health index based on the multi-pollutant CGAIM; CGAIM, constrained groupwise additive index model; NO_2, nitrogen dioxide; O_3, ozone; $PM_{2.5}$, particulate matter with aerodynamic diameter ≤ 2.5 μm; SO_2, sulfur dioxide.
